# On the Measurement of Ecological Novelty: Scale-Eating Pupfish Are Separated by 168 my from Other Scale-Eating Fishes

**DOI:** 10.1371/journal.pone.0071164

**Published:** 2013-08-19

**Authors:** Christopher H. Martin, Peter C. Wainwright

**Affiliations:** Department of Evolution and Ecology and Center for Population Biology, University of California Davis, Davis, California, United States of America; University of Lausanne, Switzerland

## Abstract

The colonization of new adaptive zones is widely recognized as one of the hallmarks of adaptive radiation. However, the adoption of novel resources during this process is rarely distinguished from phenotypic change because morphology is a common proxy for ecology. How can we quantify ecological novelty independent of phenotype? Our study is split into two parts: we first document a remarkable example of ecological novelty, scale-eating (lepidophagy), within a rapidly-evolving adaptive radiation of *Cyprinodon* pupfishes on San Salvador Island, Bahamas. This specialized predatory niche is known in several other fish groups, but is not found elsewhere among the 1,500 species of atherinomorphs. Second, we quantify this ecological novelty by measuring the time-calibrated phylogenetic distance in years to the most closely-related species with convergent ecology. We find that scale-eating pupfish are separated by 168 million years of evolution from the nearest scale-eating fish. We apply this approach to a variety of examples and highlight the frequent decoupling of ecological novelty from phenotypic divergence. We observe that novel ecology is not always tightly correlated with rates of phenotypic or species diversification, particularly within recent adaptive radiations, necessitating the use of additional measures of ecological novelty independent of phenotype.

## Introduction

Novel ecology, independent of any phenotypic novelty, is rarely adequately addressed in discussions of evolutionary novelty, except in the very general sense of an increase in niche diversity ([1]–[4]; but see [5]–[9]). Here we define novel ecology as the adoption of resources and a way of life (sensu Simpsons's adaptive zones [10]) not only unique within a given community (i.e. niche diversity), but often unique across a clade's global range and discontinuous with global niche diversity within that clade (Table 1). For example, only a single species of spider is known to be herbivorous, feeding on the Beltian bodies of an ant-plant [11]. Likewise, blood-feeding, folivory, and tool-use are unique to Darwin's finches, despite the global abundance of these resources in all passerine communities [12], [13]. Nonetheless, our perception of the novelty of any particular niche is still dependent on the size of the outgroup used for comparison and thus remains subjective (e.g. [5], [9], [14]).

**Table 1:** In most classic examples of adaptive radiation in isolated, competitor-reduced environments, a few species have invaded novel ecological niches in which they exploit omnipresent resources for the first time relative to niche use within their much larger paraphyletic outgroup.

	novel niches within adaptive radiation	outgroup niche use	references
Darwin's finches	blood and parasite-feeder, folivore, tool-using wood-probing insectivore, cactus-feeder, warbler-like insectivore	**all other domed-nest tanagers**: granivores, nectar-feeders	[12], [13]
Hawaiian honeycreepers	wood-probing insectivores, including beetle larvae and weevil specialists, cross-billed caterpillar specialists, host-specialized nectar-feeders, frugivores, insectivores	**Cardueline finches**: granivores	[86], [87], [97]
Cuban *Anolis* lizards	Twig-giant facultative molluscivores as juveniles	**all other ** ***Anolis***: arboreal, stream, and terrestrial insectivores, rarely consuming molluscs	[9], [98]
haplochromine cichlid fishes in Lakes Malawi and Victoria	scale-eaters, fry-stealing specialists, ambush and pursuit piscivores, zooplanktivores, shrimp-eaters, sand-sifters, parasite-feeders	**all other haplochromine cichlids**: algivores, detritivores, and omnivores	[29], [99]
Lake Baikal sculpin	fully pelagic viviparous amphipod-feeders, deep-water specialists	**other freshwater sculpin (** ***Cottus*** **)**: shallow-water benthic omnivores	[100], [101]
Lake Baikal amphipods	pelagic mysidiform, brood parasites, egg parasites, burrowers, free-swimming predators with extensive gigantism and sexual dimorphism	**all other freshwater amphipods (** ***Gammarus*** **)**: benthic detritivores	[102], [103]
Hawaiian Drosophilidae	larval specialists on spider eggs, flowers, leaves, roots, stems, bark, tree sap, leaf-miners	**continental Drosophilidae**: larval specialists on fruits, fungi, plants	[104]–[106]
Hawaiian *Tetragnatha* spiders	web-less pursuit hunters, web-builders using new habitats in canopy and forest floor	**continental ** ***Tetragnatha***: riparian web-builders	[107]–[109]
Hawaiian silverswords	monocarpic and polycarpic rosette plants, trees, shrubs, lianas, cushion plants, mat plants	**California tarweeds**: annual and perennial herbs	[22], [110]
Guianan *Brocchinia* bromeliads	insect carnivores, myrmecophytes, trees, mutualist with nitrogen-fixing cyanobacteria, fire-resistant species	**other bromeliads**: tank-forming epiphytes, terrestrial bushes	[5]

Note that only novel niches within adaptive radiations are listed (in nearly all cases, niche diversity observed in outgroups is also contained in adaptive radiations).

There is currently no index for quantifying the rarity or novelty of an ecological niche within a clade, to our knowledge, despite the extensive literature on quantifying niche diversity (e.g. [15]–[20]). While approaches such as niche modeling have proven incredibly productive for measuring ecology [19], [21], it is easy to overlook discontinuous resource use (i.e. novelty) when examining only shared resource axes among taxa (e.g. [6]). Thus, at the macroevolutionary level, ecological novelty is only described qualitatively [5], [9], [10], [14] or phenotypic diversification is used as an indicator of ecological diversification (e.g. [22], [23]), despite the fact that these axes of organismal diversification are sometimes decoupled (e.g. [5], [24]–[28]).

Foraging on the scales of other fishes is a specialized predatory niche surprisingly rare across the teleost tree of life despite the omnipresence of this resource in all fish communities. Specialized scale-eating has evolved at least 19 times independently (4–6 times in both African cichlids and South American characoids, plus examples in four additional freshwater families and seven marine families [29]–[32]) and is currently known in about 50 species of teleost [30] and the cookie-cutter shark (*Isistius brasiliensis*). Scale-eating is accomplished through a wide variety of morphological and behavioral solutions, including open-gaped ram feeding [33], one-sided lateral strikes with asymmetrical jaws or behavioral handedness [31], [34]–[37], aggressive mimicry, rasping with external teeth, group hunting, cleaning and mucus-feeding (reviewed in [30]), and possibly deep-water pelagic ambush [32]. Tooth shape is also exceptionally variable among scale-eaters, even within scale-eating clades [30], [38].

Despite the considerable diversity of morphological and behavioral strategies that underlie the scale-eating trophic specialization, there appear to be some universal features of this ecological strategy. First, all scale-eaters must be small relative to the size of their prey due to the high energy-to-resource ratio per strike [30]. For example, juvenile facultative scale-eaters switch to piscivory after they grow larger than their prey [30]. Conversely, the most specialized scale-eaters often switch completely to scales when they reach adult size and never grow larger than their prey [31], [33]. Second, a corollary to this observation is that specialized scale-eaters never forage on both scales and whole fish (other than fish larvae, e.g. [39]) at the same time. Third, scale-eater populations always remain much smaller than their prey populations. Lastly, lateral jaw asymmetry has evolved in scale-eating specialists at least four times independently across a wide range of foraging strategies and habitats (from Amazonia to Lake Tanganyika to the mesopelagic ocean: [31], [32], [34], [36]; but also see [40]). This suggests that laterally asymmetric jaws may be a universally adaptive trait for scale-eaters by allowing lateral attacks while pursuing prey, whereas symmetrical jaws may require perpendicular alignment with the prey. The many scale-eating specialists with symmetric jaws (including the scale-eating pupfish) may be constrained by negligible genetic variation for jaw asymmetry.

Across the global distribution of approximately 1,500 species of atherinomorph fishes [41], no scale-eaters have been documented previously (see review in [7]). We investigated reports of a potential piscivore or scale-eater within a 10,000-year-old adaptive radiation of *Cyprinodon* pupfishes (Atherinomorpha: Cyprinodontidae) endemic to a single 11-mile long island in the Bahamas [42]–[44]. The spectacular natural history of the scale-eating pupfish inspired us to adopt a simple phylogenetic novelty index to quantify ecological novelty on a temporal scale: evolutionary distance to the most closely related species with convergent ecology. Our approach was inspired by several case studies of morphological novelties which extoll the novelty of a trait relative to the age of the clade from which it has emerged [45]–[49].

Here we document the rapid evolution of lepidophagy within this radiation and test for three convergent features of the scale-eating niche: 1) reduced adult size relative to prey, 2) absence of piscivory, and 3) low frequency relative to prey population. We then apply the phylogenetic novelty index to a variety of examples of ecological novelty in order to place our discovery of a scale-eating pupfish in context. This is an example of a dramatic ecological transition that is largely decoupled from phenotypic divergence, and like other similar examples, has thus been previously overshadowed by a focus on phenotype as a proxy for ecology. We propose further that many recent adaptive radiations contain a previously overlooked dimension of diversification: ecological novelty (Table 1). We identify a 168-million-year ecological novelty within a recent adaptive radiation of *Cyprinodon* pupfishes, use a phylogenetic-distance metric to quantify this novelty, and find that the ecological novelty index within this radiation far exceeds that of a second *Cyprinodon* adaptive radiation with nearly 3-fold higher rates of morphological diversification. Our study addresses three major questions throughout: 1) Do clear examples of ecological novelty exist within extremely young species? 2) Can ecological novelty be measured independent of phenotype? 3) Is ecological diversification always strongly associated with morphological diversification?

## Methods

### Lepidophagy

We broadly define lepidophage as any free-living, pursuit or ambush aquatic predator which non-lethally removes scales, skin, mucus, fins, eyes, or whole chunks of its prey (e.g. [29], [30]). We use the term ‘scale-eater’ for this niche, although no scale-eater derives nutriment exclusively from scales, but rather the protein-rich mucus and skin tissue surrounding scales (e.g. [50]). There are no obligate scale-eaters (but see one example of an obligate mucus-feeder: [51]); however, when a species spends the majority of its foraging time extracting scales from other fishes it generally shows specialized morphology or behaviors which are presumably adaptations for scale-eating. Thus, as with previous authors [30], we distinguish specialized scale-eaters from the many species which incidentally forage on scales removed during aggressive bouts, attempts at piscivory, or scavenged from dead fishes (e.g. [52], [53]).

### Sampling

We investigated reports of a “piscivorous form” [43] or a “scale-eater/piscivore” [44] within a recent adaptive radiation of *Cyprinodon* pupfishes endemic to San Salvador Island, Bahamas. This undescribed species (*Cyprinodon* sp. ‘bulldog’) is confined to several interior hypersaline lakes on the island in sympatry with two closely related *Cyprinodon* species (*C*. sp. ‘normal’ and *C*. sp. ‘durophage’ [7]) and only two other euryhaline fish species (*Gambusia hubbsi* and *Atherinomorus stipes*; [43], [44]). *C*. sp. ‘normal’ is nearly indistinguishable in morphology and habit to widespread Caribbean and Atlantic coast populations of *C. variegatus*. *C*. sp. ‘durophage’ possesses a novel nasal appendage formed from a skeletal extension of the head of the maxilla which may facilitate hard-shelled prey extraction ([7], CHM pers. obs.).

This radiation of three sympatric *Cyprinodon* species shows significant genetic differentiation within and among lakes (*F_st_* = 0.1–0.31; [44]). Despite ongoing gene flow, as in many recent adaptive radiations (e.g. [54]), all three species are partially reproductively isolated due to strong ecological selection against hybrids with intermediate phenotypes [55]. Initial field observations and laboratory trials also indicate that *C*. sp. ‘bulldog’ is further isolated by female mate choice for conspecific males [CHM pers. obs.]. Preliminary genomic-scale analyses of genetic structure among these species support a single origin of each specialist species followed by dispersal among lakes [CHM unpublished data]. Our initial results also indicate that *C*. sp. ‘bulldog’ individuals form a monophyletic clade across lakes, indicating strong reproductive isolation from *C. variegatus* and *C*. sp. ‘durophage’ [CHM unpublished data]. *C*. sp. ‘bulldog’ and *C*. sp. ‘durophage’ are currently being formally described as new species [Martin and Wainwright in revision].

In July 2008, all three *Cyprinodon* species were collected from two lake populations, Crescent Pond (CP) and Little Lake (LL), on San Salvador Island, Bahamas. Adults were sampled from 0.3–2 m depth using a hand net while snorkeling or by seine net. At the surface, individuals were immediately euthanized in an overdose of MS-222 (Finquel, Argent Laboratories Inc.). Animal care procedures followed the recommended guidelines for laboratory animal care and were approved by the University of California, Davis Animal Care and Use Committee (Protocols #15640, 15908). Further digestion was halted with an intra-peritoneal injection of 15% formalin, followed by fixing the whole specimen in 15% formalin. After fixing, specimens were moved to 70% ethanol for long-term storage. Research permits were approved by the Bahamas Environment, Science & Technology commission, Ministry of the Environment, and the Ministry of Agriculture (2011); and by the Department of Marine Resources, Ministry of Agriculture and Marine Resources (2008) with the support of the Gerace Research Centre.

In the laboratory, approximately 3 cm of the anterior gut was removed from each individual for stomach content analysis. Food items were spread on a Sedgwick-Rafter cell and sorted at 25× magnification under a stereoscope. Items were identified to class with additional categories for scales, whole fishes (comprising both *Cyprinodon* and *Gambusia* species), detritus, and crushed shells or silt. Major food items included various macroalgae (predominantly *Batophora oerstedi* and *Cladophoropsis macromeres*), widgeon grass (*Ruppia maritima*), ostracods, various gastropods, scales, whole fish, and polychaetes (Table 2). The total volume of each dietary component was estimated from the total number of 1 µl cells covered in the chamber. The proportion of each dietary component was calculated from its volume relative to the total volume of all components for each individual (as in [24]; Table 2). Individuals with empty stomachs (*n* = 22) were excluded.

**Table 2:** Proportional stomach contents (mean ± SE) of *Cyprinodon* pupfishes from San Salvador Island, Bahamas.

	*Cyprinodon* sp. *‘bulldog’*		*C.* sp. *‘durophage’*		*C.* sp. *‘normal’*	
	CP	LL	CP	LL	CP	LL
Item	*n* = 25	*n* = 28	*n* = 32	*n* = 28	*n* = 22	*n* = 41
**scales**	**.51±.06**	**.40±.06**	.001±.00	.01±.01	.001±.00	.001±.00
whole fish	-	-	-	-	-	**.08±.04**
macroalgae	.10±.06	.02±.01	.03±.03	.05±.02	**.13±.06**	**.20±.05**
plant matter	.00004±.0	.003±.00	.05±.02	.01±.01	**.09±.04**	**.05±.03**
gastropod	.10±.06	.01±.00	**.10±.04**	**.22±.06**	.01±.01	.04±.02
bivalve	-	-	-	.0004±.00	-	.02±.01
ostracoda	-	.004±.00	**.30±0.05**	.04±.03	0.001±.00	.0003±.00
amphipoda	-	-	-	-	.0005±.00	.01±.01
odonata	-	-	-	-	-	.02±.02
arthropoda (unidentified)	.0003±.00	.01±.00	.02±.01	-	.005±.005	.002±.00
polychaeta	-	.03±.01	-	.0002±.00	.05±.04	.04±.01
detritus	.29±.05	.49±.06	.50±.06	.57±.08	.71±.08	.34±.05
silt/shells	-	.02±.01	-	.10±.04	-	.20±.05

CP = Crescent Pond population; LL = Little Lake population. Major food items are bold-faced for each species in each population (ignoring detrital content). Individuals with empty stomachs were excluded (*n* = 22).

Visual censuses of species abundance were also conducted in three lakes containing all three *Cyprinodon* species in July 2008. All adults and subadults were counted by a single observer within a 0.3 m×30-m transect while snorkeling. Censuses were repeated 4 times each in Crescent Pond, Little Lake, and Osprey Lake. Although population abundances vary seasonally and annually, these censuses estimate the relative abundance of scale-eaters and their prey.

In March and July 2011, adults (>2.0 cm) from all three species were collected from Crescent Pond and Little Lake for stable isotope analyses. After euthanasia, approximately 5 mg of muscle tissue was removed from the caudal peduncle of each specimen and dried at 60°C for 48 hours. Tissue samples were weighed and submitted to the UC Davis Stable Isotope Facility for measurement on a PDZ Europa ANCA-GSL elemental analyzer, interfaced to a PDZ Europa 20–20 isotope ratio mass spectrometer (Sercon Ltd., Cheshire, UK). For comparisons of standard length (SL), adults of each species from larger samples collected in 2008 and 2011 were measured using dial calipers.

We also performed a literature search to identify all Cyprinodontidae species with published data on stomach contents (reviewed in [7]). Additional species descriptions, field guides [56], [57], data from FishTraits [58], and community knowledge (e.g. American Killifish Association) confirmed that no other atherinomorphs are known scale-eaters.

### Phylogenetic novelty index

Ideally, the rarity of any ecological niche within a clade could be estimated from the transition rate among niche states across a distribution of time-calibrated phylogenies with near-complete sampling at the species level (e.g. see [59] for transition rates among dietary categories in 1/3 of all mammal species). This approach would incorporate much of the uncertainty in phylogenetic estimation and time-calibration (assuming adequate prior estimates of node ages) by summing over a posterior distribution of models and phylogenies [60], [61]. However, studying rare ecological transitions across the tree of life is still limited by the lack of large trees with near-complete sampling at the species level (or even large trees with 50% sampling, as investigated by [62]). Thus, it is rarely possible to find one chronogram containing multiple species with rare, convergent ecology.

Due to the current lack of large chronograms connecting rare convergent ecology, we used parsimony to reconstruct the origin of rare ecological niches. Parsimony ignores uncertainty in ancestral estimation of niche evolution (e.g. [63]); however, this uncertainty is relatively minor for rare, recent events across the tree of life (e.g. blood-feeding most likely evolved within the adaptive radiation of Darwin's finches). Furthermore, parsimony is not sensitive to incomplete lineage sampling (e.g. [64]).

**Figure 1:**
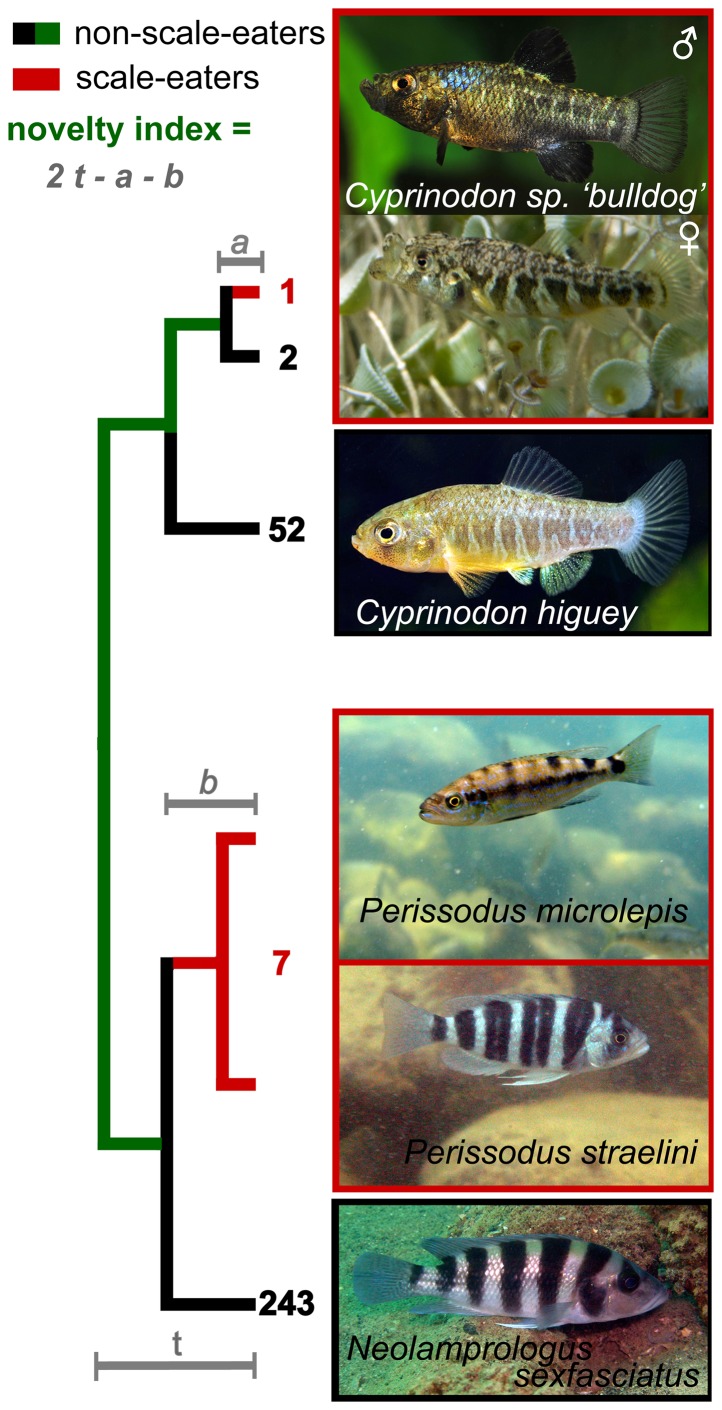
Illustration of the phylogenetic novelty index applied to scale-eating in *Cyprinodon* sp. ‘bulldog’. The most closely related species with convergent ecology are a clade of scale-eating cichlids from Lake Tanganyika, *Perissodus* spp. A simplified cladogram connecting these groups is illustrated with numbers at tips corresponding to the number of scale-eating (black) and non-scale-eating (red) species within the *Cyprinodon* and Tanganyikan haplochromine clades (note that thousands of additional outgroup species have been pruned and these species numbers are not presented). Phylogenetic novelty index (indicated by the green line; 168 million years in Table 3) is calculated from twice the divergence time (*t*) minus the estimated origin of scale-eating in each clade (*a* and *b*). The stem age of the *Perissodus* clade is used as a conservative estimate of the origin of scale-eating (*b*). Note that the phylogenetic novelty index is not the same if applied to scale-eating cichlids (Table 3), which have repeatedly colonized this niche within each Great Lake radiation. Also note the aggressive mimicry in *Perissodus straelini* and the crypsis of female *Cyprinodon* sp. ‘bulldog’. Photo credits: Jennifer O. Reynolds, Tony Terceira.

We calculated phylogenetic novelty from the amount of time separating two lineages with convergent ecology minus the inferred origination times of the convergent niche in each clade:
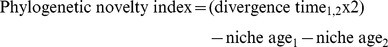
where _1_ and _2_ correspond to the most closely related species or clades exhibiting convergent ecology (Fig. 1). Thus, novelty is measured as the time over which ancestors connecting two convergent ecological niches do not occupy the niche. This timespan should be inversely correlated to the probability of re-emergence of the niche which may fall off exponentially with increasing time, as expected under a diffusion process of continuous trait evolution (e.g. [65]). Unlike transition rates calculated from discrete character shifts across a tree [59], [66], [67], this temporal measure of novelty is unaffected by relative lineage diversification rates among the groups with convergent ecology.

Note that this index is not symmetric due to the hierarchical structure of phylogenies. Thus, scale-eating pupfish are equally distant from all cichlids, in which scale-eating has evolved multiple times, whereas scale-eating cichlids are more closely related to other lineages of scale-eating cichlids and exhibit a much lower novelty index (Table 3). This asymmetric property of the index usefully reflects the greater novelty of scale-eating within Cyprinodontiformes than within Cichlidae.

**Table 3:** Application of the phylogenetic novelty index to examples of ecological novelty.

focal clade	focal ecology (a: niche age*)	nearest species/clade with convergent ecology (b: niche age*)	divergence time* (t)	novelty index* (2t-a-b)	references
San Salvador *Cyprinodon* pupfishes	**scale-eater**: *C*. sp. ‘bulldog’ (0.005–0.015‡)	Tanganyikan *Perissodus* spp. cichlids (1.5–2.2)	85	168	[44], [73], [77]
Lake Chichancanab *Cyprinodon* pupfishes	**piscivore**: *C. maya* (0.008‡)	*Orestias cuvieri* (0.06–1.5‡)	21†	40	[7], [111]–[113]
	**zooplanktivore**: *C. simus* (0.008‡)	*Aphanius anatoliae splendens* (5–10‡); *Orestias ispi* (0.06–1.5‡)	21†	32	
Darwin's finches	**blood-feeder**: *Geospiza difficilis septentrionalis* (2–3‡)	oxpecker *Buphagus* spp. (20–23)	23	20	[12], [114], [115]
	**seed-eater**: *Geospiza* spp. (2–3‡)	Cocos Island finch *Pinaroloxias inornata* (1.9–2.4‡)	2–3‡	0	
*Anolis* lizards	**twig-giant molluscivore**: *Anolis (Chameleolis)* spp. (<15–20†)	*Diplolaemus bibroni* (?)	145	<270	[116], [117]
Guianan *Brocchinia* bromeliads	**carnivory**: *Brocchinia reducta* (<9)	*Catopsis berteroniana* (<14)	19	<15	[5], [118]
	**ant-plant**: *Brocchinia acuminata* (<9)	*Tillandsia butzii* (<4)	19	<26	
*Bagheera kiplingi*	**herbivore/ant-plant parasite** (?)	Early Devonian arthropods† (398–416: first chelicerate fossil)	398–416	<398	[11], [119]
Sawfishes (Schlerorhynchidae and Pristidae)	**piscivores with elongated, serrated rostrum**: (130†)	Sawsharks (Pristiophoriformes) (72-66†)	251–374	300	[120]–[122]
Tanganyikan *Perissodus* scale-eating cichlids	**scale-eater:** Tanganyikan *Perissodus* spp. (1.5–2.2)	Malawi cichlids: *Genyochromis mento*, *Corematodus* spp. (<1–2); Victorian cichlid *Haplochromis welcommei* (<0.1)	24	44	[29], [77], [123]
Paxton Lake benthic stickleback	**Benthivore**: (0.01‡)	Enos Lake benthic stickleback (0.01‡)	0.01	0	[91]

The novelty index is calculated by subtracting the niche ages of the focal ecology (a) and the most closely related lineage or clade with convergent ecology (b) from the time to the most recent common ancestor of the two convergent ecologies (divergence time, t) times 2 (the amount of time separating the two lineages). Lineage/clade ages and divergence times (in millions of years) were drawn from multiple chronograms, minimum age estimates from the fossil record (†), and geological ages of lake basins (‡) and should therefore be interpreted cautiously. To be conservative, we used ages which minimized the novelty index.

* Units in millions of years.

† Minimum divergence time or lineage/clade age estimated from earliest known fossil.

‡ Lineage/clade age estimated from geological age of lake basin or archipelago which contains the adaptive radiation.

? Lineages with no available age information from time-calibrated phylogenies or fossils.

Complete knowledge of the most closely related species with convergent ecology (and therefore complete knowledge that all intervening species do not express the ecology) and a time-calibrated phylogeny connecting these species is necessary to calculate the novelty index. For common ecological niches this is a daunting task; however, the occurrence of highly unusual ecologies is usually much better known due to their noteworthy status. The identification of convergent ecology is still a subjective choice by the investigator which requires detailed knowledge of the natural history of the group and can only be decided on a case-by-case basis. Although identifying specialized scale-eating within pupfishes was straightforward, assignment of specialized ecology is more subjective when the use of a novel resource varies across populations (e.g. the vampire finch), similar niches can be grouped (e.g. is eye-biting a form of scale-eating or a different niche? [29]), or different species exploit the same resource in different ways (e.g. sawfish are piscivores [68]).

Due to the lack of a comprehensive chronogram containing multiple ecologically novel species, we pieced together node ages from different chronograms to apply this index to the examples in Table 3. This is not ideal and can introduce considerable bias when comparing node ages estimated from different datasets, particularly given the known discrepancies among fossil calibration points [69], [70], uncorrelated relaxed-clock models estimated independently among different taxa and genes [71], and often complex relationship among conflicting time-calibration priors, tree topology, and branch lengths (e.g. see supplemental methods to [7]). In cases where chronograms were lacking, we used fossil age estimates from the earliest known representative of a given clade or geological age estimates of the lake basin which contained the adaptive radiation (Table 3). Thus, our current estimates of the phylogenetic novelty index should be interpreted cautiously as they are dependent on the particular set of chronograms and fossils available. These estimates should serve mainly to illustrate application of our approach. Nonetheless, the exponential expansion of molecular sequencing (e.g. [72]) and trend toward increasing tree sizes with greater access to fossil calibrations [59], [73]–[75] should facilitate greater use of this index in the near future. Timetree also provides a useful database for searching existing chronograms [76].

To be conservative in calculating niche age, we took the stem age, rather than the crown age, of clades in which all species exhibit the focal ecology, such as the Tanganyikan *Perissodus* clade of scale-eaters [77], because the novel ecology likely evolved sometime along this stem lineage before the evolution of the crown group [78]. For clades in which only one species colonized the novel niche during adaptive radiation, such as San Salvador pupfishes, we took the crown age of the radiation for the niche age because the novel niche was likely colonized sometime along this terminal branch within the radiation. However, formal ancestral reconstruction methods (e.g. [63]) for estimating a range of times for the origin of novel ecology would be more appropriate for more recently diverged taxa or when clade membership is uncertain. Formal ancestral state reconstruction methods allow inference of niche origination times across a distribution of trees incorporating phylogenetic uncertainty [60]. Nonetheless, most of the uncertainty in estimation of the phylogenetic novelty index arises from the large variance in estimates of node age, even when comparing nodes ages estimated jointly within the same chronogram. Increasing sophistication of time-calibration methods (e.g. [79]) and increased incorporation of fossil data should greatly improve inference of clade ages.

We include common ecological niche transitions for comparison in Table 3; however, we stress that the use of parsimony for ancestral character reconstruction is never justified when considerable uncertainty is present and likelihood or Bayesian estimation of ancestral character states is needed. In practice, as a given niche becomes more commonly used across a clade (i.e. as niche transition rate increases), the phylogenetic novelty index rapidly goes to zero as uncertainty in reconstruction of ancestral states prevents robust inference of ancestral niche use. Common niche use also makes assignment of convergent ecology more subjective. Instead, we suggest this index is most useful for quantifying and comparing rare niche transitions which can be treated as discrete.

One weakness of this approach, common to all comparative phylogenetic analyses based only on extant taxa, is the lack of ecological knowledge of extinct lineages which may have also colonized the focal niche. This biases estimates of phylogenetic novelty upwards. In particular, very large estimates of phylogenetic novelty should be regarded with increased suspicion due to the greater probability of extinct lineages occupying the focal niche. However, if ecological inferences about extinct taxa are available, this information can easily be incorporated into the novelty index. For example, including fossil sawsharks and sawfishes substantially reduces the ecological novelty of this specialized mode of foraging relative to comparisons of only extant sawfish and sawsharks by increasing estimates of the origins (niche age) of this convergent foraging strategy within each lineage (Table 3).

## Results

### Lepidophagy

**Figure 2:**
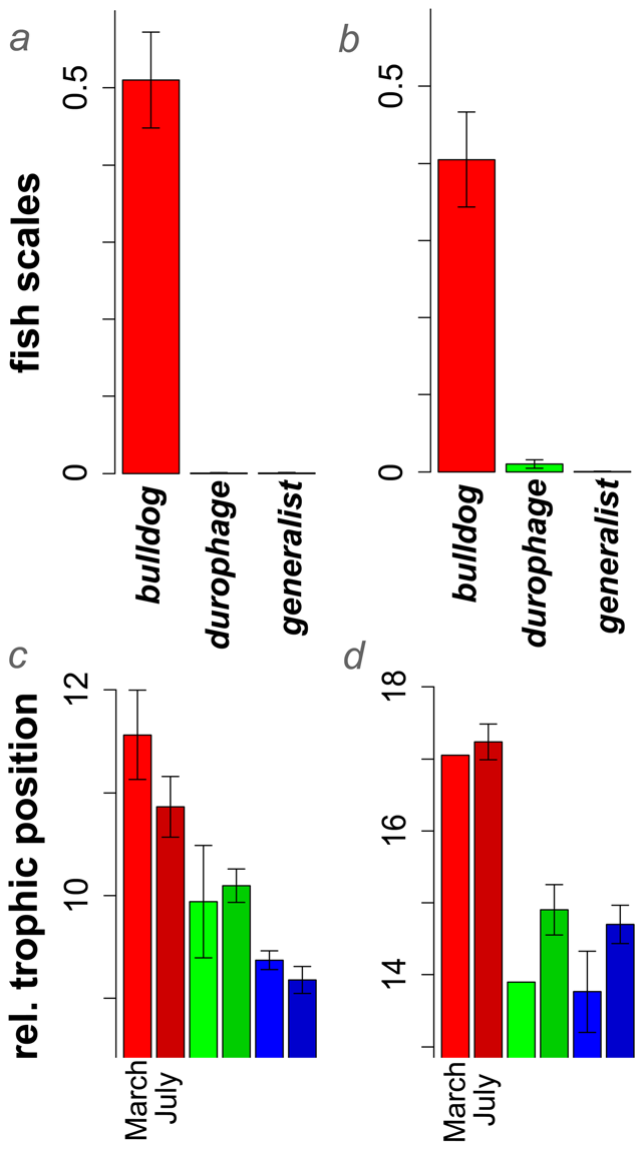
Diets of *Cyprinodon* sp. ‘bulldog’ (red), C. sp. ‘durophage’ (green), and C. sp. ‘normal’ (blue) in two hypersaline lakes on San Salvador Island, Bahamas. *a,b)* Proportion of scales (mean ± SE) in stomach contents of each species in *a)* Crescent Pond and *b)* Little Lake populations. *c,d*) Relative trophic position (δ^15^N: mean ± SE) of each species from samples collected in March (first bar) and July (second bar) in *a)* Crescent Pond and *b)* Little Lake populations. Multiple samples of ‘bulldog’ and ‘durophage’ were not available in March in Little Lake.

40–51% of stomach contents of *Cyprinodon* sp. ‘bulldog’ were comprised of scales from other *Cyprinodon* and possibly *Gambusia* fishes in two lakes (Fig. 2, Table 2). Not a single bulldog individual had ingested whole fish, despite the occurrence of piscivory in *Cyprinodon* sp. ‘normal’ (Table 2). Stable isotope analyses confirmed that *C*. sp. ‘bulldog’ occupied the highest trophic position (δ^15^N) relative to sympatric *Cyprinodon* species in both winter and summer seasons in two lakes (Fig. 2).

Scale-eating by *C*. sp. ‘bulldog’ was frequently observed in the wild and occurred approximately once per minute during daylight hours (CHM pers. obs.). Adult *C.* sp. ‘bulldog’ were slightly smaller (CP: 2.27±.05 (mean ± SE; *n* = 17), LL: 2.68±.04 cm SL (*n* = 21)) than their most common prey target, *C*. sp. ‘normal’ (CP: 3.08±.07 (*n* = 39), LL: 3.01±.06 cm SL (*n* = 62)). *C.* sp. ‘normal’ was, by far, the most abundant species in all three lakes surveyed (mean % ± SE: CP: 92.7±1.0; LL: 92.3%±1.1; OL: 93.8%±1.7). *C*. sp. ‘bulldog’ frequency ranged from 0.2–3.1% of all pupfishes across the three lakes surveyed (CP: 0.9±0.4; LL: 3.1±0.8; OL: 0.2±0.1).

### Phylogenetic novelty index

The most recent maximum clade credibility ultrametric tree for Ovalentaria places cichlids + *Pholidichthys* + Polycentrids as the sister group to atherinomorphs [73], [74]. This node is weakly supported and blennioids may also be the most closely related group containing a scale-eating species, the mimic fangblenny (*Plagiotremus tapeinosoma*); however, this alternative phylogenetic hypothesis has little impact on the estimated novelty index. Under the phylogenetic hypothesis of cichlids + *Pholidichthys* + Polycentrids as sister group to atherinomorphs, the most closely related specialized scale-eaters to *Cyprinodon* sp. ‘bulldog’ are found in several lineages of scale-eating cichlids within adaptive radiations in Lakes Victoria, Tanganyika, and Malawi [29], [77]. Using the stem age of the oldest of these scale-eating cichlid clades, the Tanganyikan *Perissodus* clade [77], we estimate that approximately 168 million years separates the San Salvador scale-eating pupfish from the origins of scale-eating in the *Perissodus* stem lineage (Fig. 1, Table 3). Several classic examples of adaptive radiation display astonishing levels of ecological novelty by this index (e.g. the vampire finch, the false Chameleon *Anolis*: Table 3), far exceeding their phenotypic novelty (e.g. [13]). Conversely, classic radiations which only partition a subset of their ancestral resource base do not show unusual levels of ecological novelty due to frequent parallel evolution of the same niche across similar environments (e.g. threespine sticklebacks, *Geospiza* seed-eating groundfinches, Chichancanab pupfishes: Table 3).

## Discussion

We document the recent evolution of a scale-eating pupfish, a unique niche within over 1,500 atherinomorph species distributed globally (Fig. 2, Table 2). To place this novelty in perspective, we observe that the most closely related scale-eating specialist is separated by 168 million years of evolution from the scale-eating pupfish (Fig. 1). We propose this simple phylogenetic novelty index as a quantitative measure for comparing rare and novel ecological transitions across the tree of life and apply it to several classic and unusual niches (Table 3). This index generalizes estimates of novelty beyond the bias of restricting attention to a named clade or outgroup (e.g. Table 1; [47]) and complements qualitative descriptions of ecological novelty (e.g. [5], [9], [10]). Although identifying convergent ecology often remains subjective on a case-by-case basis, our approach provides a method for quantifying the rarity of unassailable examples of convergent ecology across the tree of life.

### Lepidophagy

We document the evolution of a specialized scale-eating pupfish species, *Cyprinodon* sp. ‘bulldog’, within a sympatric adaptive radiation on San Salvador Island, Bahamas. 41–51% of its diet was composed of scales (Fig. 2, Table 2). The actual proportion was probably much higher as most of the remaining stomach contents in this species were detritus, which may consist mainly of digested skin and mucus tissue from scale-feeding (Table 2). Scale-eating was independently supported by the higher trophic position of *C*. sp. ‘bulldog’ across lakes and seasons as inferred from δ^15^N isotope ratios (Fig. 2). As predicted by the functional and ecological constraints on specialized scale-eating, *C*. sp. ‘bulldog’ was slightly smaller than its prey, never captured whole fish, and occurred at low frequency across all three populations surveyed.

Rates of morphological evolution in the San Salvador *Cyprinodon* radiation are exceptional outliers among *Cyprinodon* clades [7]. For example, jaw length and adductor mandibulae muscle mass are diversifying 51 and 47 times faster than background rates in allopatric species, respectively [7]. In a second, independent adaptive radiation of *Cyprinodon* pupfishes endemic to Laguna Chichancanab, rates of morphological diversification are two- to three-fold higher: tooth length and adductor muscle mass are diversifying 131 and 120 times faster than background rates, respectively [7]. These exceptional rates of morphological diversification are not only due to the young age of these clades, but rather the colonization of novel ecological niches: scale-eating and durophagy on San Salvador Island, Bahamas and piscivory and zooplanktivory in Laguna Chichancanab, Mexico [7].

We show that the ecological novelty of the San Salvador adaptive radiation is even more exceptional than its morphological diversification rate. A single species within this clade has recently colonized an ecological niche, scale-eating, that is approximately 168 million years removed from the most closely related species with convergent ecology (Fig. 1, Table 3). The phylogenetic novelty index does not account for extinct scale-eating taxa; however, the rarity and complete absence of this feeding specialization outside lacustrine adaptive radiations in atherinomorphs suggests this may be an accurate estimate of the novelty of this niche. Although the Laguna Chichancanab *Cyprinodon* radiation is diverging nearly three-fold faster for certain morphological traits, these species have invaded ecological niches that are not particularly novel for Cyprinodontidae, piscivory and zooplanktivory. Piscivory is common within nearly all fish groups and the most closely related specialized piscivore is most likely *Orestias cuvieri*, the extinct top-predator from the Lake Titicaca adaptive radiation of *Orestias* pupfishes [80]. Specialized zooplanktivores are also known from the Lake Titicaca radiation (e.g. *O. ispi* [81]) and Old World *Aphanius* pupfishes such as *Aphanius anatoliae splendens* [82]. The phylogenetic novelty index highlights the exceptional ecological novelty of a scale-eating pupfish within the San Salvador radiation, despite the nearly 3-fold lower morphological diversification rates in this clade (Table 3).

### On the evolution of ecological novelty

Application of the phylogenetic novelty index to several recent and classic examples of adaptive radiation (Table 3) illuminates dramatic examples of extremely rare, major ecological transitions which are easily overlooked when using morphological proxies for ecology. For example, the beaks of Darwin's finches are not particularly diverse relative to other domed-nest clade finches in the Caribbean [13]. However, the vampire finch (*Geospiza difficilis septentrionalis*) has adopted a novel and extremely rare niche among birds: blood-feeding. This resource forms a major part of the diet in only one other specialized lineage of birds, the oxpeckers (*Buphagus* spp.), which are also known to open wounds and drink blood [83], [84]. Bill shapes of the vampire finch and other ecologically novel Darwin's finches, such as the vegetarian finch and tool-using woodpecker finch, are not particularly unusual [12], [13]. Conversely, the widely divergent beak shapes of Darwin's seed-eating ground finches (e.g. *Geospiza magnirostra* vs. *Geospiza fuliginosa*) correspond to specializations for eating seeds of various sizes [12], a very common niche across the domed-nest clade ([13]; Table 2). Thus, Darwin's finches provide an excellent example of the decoupling of ecological novelty from dramatic morphological diversification.

Once we are able to measure differences in ecological novelty among taxa, the highly uneven distribution of ecological novelty among clades and environments begs explanation. Ecological novelty does not appear to be distributed randomly among clades, but often reappears repeatedly within the same clades invading similar environments and may be particularly common within recent adaptive radiations (Table 1). Why are some populations able to exploit these novel resources but not others? Are transitions to novel niches necessary for population persistence or would partitioning more common resource axes be sufficient? What combination of environment and lineage-specific factors is necessary to drive the evolution of novel ecology? These questions are particularly clear when comparing similar lineages that have speciated and adapted to the same environment, but display exceptional differences in their propensity to evolve ecological novelty: for nearly every compelling example of island adaptive radiation, there are similar lineages that have colonized the same environment and speciated to some extent, but fail to evolve novel ecology, such as finches and mockingbirds in the Galapagos [12], [85], honeycreepers and thrushes in the Hawaiian Islands [86], [87], pupfishes and mosquitofish in Caribbean salt lakes [7], [88], and different lineages of cichlids within sympatric radiations [89], [90]. This pattern also occurs when the same lineage is distributed across many similar environments: rapid ecological diversification is often restricted to a few places [7], [13].

The evolution of ecological novelty, a subset of many types of niche divergence between populations, remains unexplained by existing ecological divergence mechanisms (e.g. [91]); as with phenotypic diversification, we have a solid theoretical and empirical framework for microevolutionary change, but no clear link between these processes and larger patterns of macroevolutionary diversification and stasis (e.g. [92]). For example, why has such an exceptionally rare trophic niche evolved only on San Salvador Island in the Bahamas? One possibility is that adaptation to novel ecological niches is strongly influenced by the location of their corresponding fitness peaks on the adaptive landscape. Field experiments on San Salvador Island measuring the fitness landscape for thousands of F2 hybrids placed in field enclosures support this idea [55]. Two fitness peaks for growth and survival within the range of hybrid phenotypes measured corresponded to the phenotypes of *C*. sp. ‘normal’ and *C*. sp. ‘durophage’ observed in the wild. In contrast, hybrids resembling the scale-eater had low growth and survival across two lakes at two different densities [55]. If a fitness peak for scale-eating exists in this environment, it may require a highly specialized phenotype for successful performance which was not recovered within the F2 hybrids used in this experiment. Thus, rare novel niches may reflect distant, isolated, or narrow fitness peaks surrounded by a large fitness valley on the adaptive landscape.

More generally, how should the ecological novelty index be interpreted? This index is a quantitative measure of the rarity of a niche. Alternatively, with complete lineage sampling, we could estimate the transition rate into any niche from formal ancestral state reconstructions as a quantitative measure of rarity: lower transition rates correspond to rarer niches. However, with this approach, transition rates also depend on lineage diversification rates. Is a rare niche more novel within a clade of 100 species than within a clade of 10 species? We think a more relevant measure of novelty is the minimum distance to a species with convergent rare ecology, regardless of lineage diversification rates spanning this time period.

Second, the niche is a complex and dynamic mapping of population persistence onto a hyper-dimensional ecological space [20], [93] and an emergent property of both the evolving organism and the shifting biotic and abiotic environments [10], [94], [95]. The rarity of any ecological niche is a function of the global abundance of its ecological space and the abundance of taxa able to persist (‘fundamental niche’) and currently competing within that space (‘ecological opportunity’). Thus, the novelty index can also be interpreted as the ‘findability’ (see [96]) of a niche on the adaptive landscape across the global biosphere, given these constraints of abundance, persistence, and competition.

### Conclusion

While evolutionary novelty is frequently addressed at other levels of biological organization, here we provide a framework for quantifying and comparing novelty at the ecological level. We define ecological novelty as a major ecological transition to a new adaptive zone, often unique across a clade's global range (Table 1). We use a phylogenetic novelty index for quantifying ecological novelty: the time separating the inferred origin of the novelty from the inferred origin of the most closely related species with convergent ecology (Fig. 1). A synthesis of previous scattered observations reveals that ecological novelty is particularly common within recent adaptive radiations in isolated environments (Table 1) and that specialized species colonizing novel ecological niches are not necessarily the most phenotypically divergent (Table 3). In particular, we document the rapid evolution of a scale-eating specialist within an incipient adaptive radiation of *Cyprinodon* pupfishes endemic to San Salvador Island (Fig. 2, Table 2). We estimate this species is separated by approximately 168 million years of evolution from the most closely related scale-eating specialist (Table 3). The phylogenetic novelty index should facilitate further comparative analyses of novelty across the tree of life and illustrate the previously overlooked dimension of exceptional ecological diversification.
